# Simulated Analysis of Influence of Changes in H^+^-ATPase Activity and Membrane CO_2_ Conductance on Parameters of Photosynthetic Assimilation in Leaves

**DOI:** 10.3390/plants11243435

**Published:** 2022-12-08

**Authors:** Ekaterina Sukhova, Daria Ratnitsyna, Vladimir Sukhov

**Affiliations:** Department of Biophysics, N.I. Lobachevsky State University of Nizhny Novgorod, Nizhny Novgorod 603950, Russia

**Keywords:** photosynthetic CO_2_ assimilation, H^+^-ATPase, chloroplast envelope CO_2_ conductance, plasma membrane CO_2_ conductance, electrical signals, spatial heterogeneity, two-dimensional photosynthetic model

## Abstract

Photosynthesis is an important process in plants which influences their development and productivity. Many factors can control the efficiency of photosynthesis, including CO_2_ conductance of leaf mesophyll, which affects the CO_2_ availability for Rubisco. It is known that electrical stress signals can decrease this conductance, and the response is probably caused by inactivation of H^+^-ATPase in the plasma membrane. In the current work, we analyzed the influence of both CO_2_ conductance in the plasma membrane, and chloroplast envelopes and H^+^-ATPase activity on photosynthetic CO_2_ assimilation, using a two-dimensional mathematical model of photosynthesis in leaves. The model included a description of assimilation on the basis of the Farquhar–von Caemmerer–Berry model, ion transport through the plasma membrane, diffusion of CO_2_ in the apoplast, and transport of CO_2_ through the plasma membrane and chloroplast envelope. The model showed that the photosynthetic CO_2_ assimilation rate was mainly dependent on the plasma membrane and chloroplast envelope conductance; direct influence of the H^+^-ATPase activity (through changes in pH and CO_2_/HCO_3_^−^ concentration ratio) on this rate was weak. In contrast, both changes in CO_2_ conductance of the plasma membrane and chloroplast envelopes and changes in the H^+^-ATPase activity influenced spatial heterogeneity of the CO_2_ assimilation on the leaf surface in the simulated two-dimensional system. These effects were also observed under simultaneous changes in the CO_2_ conductance of the plasma membrane and H^+^-ATPase activity. Qualitatively similar influence of changes in the CO_2_ conductance of the plasma membrane and chloroplast envelopes, and changes in the H^+^-ATPase activity on photosynthesis were shown for two different densities of stomata in the simulated leaf; however, lowering the density of stomata decreased the assimilation rate and increased the heterogeneity of assimilation. The results of the model analysis clarify the potential influence of H^+^-ATPase inactivation on photosynthesis, and can be the basis for development of new methods for remote sensing of the influence of electrical signals.

## 1. Introduction

Photosynthesis is a critical process in green plants that uses light energy to convert carbon dioxide and water into simple sugars. It is necessary for the nutrition of living organisms, oxygen production and carbon dioxide elimination; particularly, the leaves, seeds and roots of green plants are the basis of food security for humanity [1,2]. Investigations of photosynthesis in plants are necessary for improving plant productivity and providing their protection from the action of stressors including excess light, drought, high and low temperatures, salinity and others.

Photosynthesis is very sensitive to the influence of adverse factors which can disturb the balance of photosynthetic processes through changes in photosynthetic parameters; CO_2_ concentration in the stroma of chloroplasts in particular can be an important target of these adverse factors [3,4]. It is known that this concentration can strongly limit the rate of photosynthetic CO_2_ assimilation in the Calvin–Benson cycle [5,6]. There are numerous stressors (among other things, high temperature [7,8], drought [9,10], soil salinity [11,12]) which limit water availability for plants and cause the closing of stomata [9,13]. It is known that this stomatal closing significantly decreases the photosynthetic CO_2_ assimilation rate (A_hv_) [14], thereby suppressing the activity of the electron transport chain [15]. This suppression can cause stimulation of the regulated dissipation of light energy in light-harvesting complexes, and can decrease the size of these complexes to reduce electron flow in the electron transport chain [15,16]; in addition, the light-dependent transportation of charges in the electron transport chain can produce reactive oxygen species [17,18] which disrupt cellular structures and biochemical compounds [19] in plant cells, causing yellowing and withering of leaves and whole plants.

The availability of CO_2_ for photosynthesis can be also limited by mesophyll resistance to carbon dioxide [20,21]. Mechanisms of mesophyll conductance of CO_2_ are interesting problems which are actively investigated. It is known that lipid membranes are relatively weakly permeable to CO_2_; carbon dioxide is considered to be mainly transported through aquaporins of biological membranes [22,23,24], because modification of the number of aquaporins can change photosynthetic CO_2_ assimilation in plants [24,25]. The activity of these aquaporins can be regulated by pH and tension of membrane [9,13]. This means that environmental factors and stress signals (e.g., electrical signals) can also have influence on the transport of CO_2_ through aquaporins in the plasma membrane and chloroplast envelopes [9]. Investigation of the role of aquaporins in photosynthetic responses to the action of stressors can be important for understanding the mechanisms of photosynthesis regulation.

The CO_2_ transport from the apoplast to the chloroplast stroma and photosynthetic assimilation can also be dependent on the conversion of CO_2_ to HCO_3_^−^, which is regulated by pH in medium [26,27]. These two forms of carbon dioxide have a different rate of diffusion through water and biological membranes [3]; additionally, the possibility of direct HCO_3_^−^ use by Rubisco in the Calvin–Benson cycle in C_3_ plants is not clear.

It is known that H^+^-ATPase in the plasma membrane is a key proton transporter which controls apoplastic and cytoplasmic pH in plant cells [28]. It can be hypothesized that this transporter can also regulate CO_2_ availability; this hypothesis is supported by our previous results. In particular, we showed that changes in CO_2_ assimilation were strongly related to shifts in the apoplastic pH [29]. The theoretical analysis also showed the influence of activity of H^+^-ATPase in the plasma membrane on the ratio of CO_2_ to HCO_3_^−^ in the apoplast [27]; this ratio can potentially regulate CO_2_ mesophyll conductance. Additionally, changes in the apoplastic and cytoplasmic pH can influence CO_2_ transport through the plasma membrane and chloroplast envelopes; this effect is based on changes in the activity of aquaporins [30]. 

This potential influence of H^+^-ATPase on CO_2_ availability, which is based on increasing the apoplastic pH and decreasing the cytoplasmic pH, can be especially important for explanation of photosynthetic regulation by electrical stress signals (action potentials, variation potentials and system potentials). These signals are induced by local irritations, propagate into intact parts of plants, and modify photosynthetic processes [31,32,33,34]. It is known that generation of these electrical signals is related to changes in H^+^-ATPase activity [31,32,34,35,36]; i.e., these changes can be a mechanism of induction of photosynthetic responses through changes in CO_2_ availability in the Calvin–Benson cycle [33]. The changes in CO_2_ mesophyll conductance caused by electrical signals and related to changes in pH [30] support the hypothesis concerning the influence of changes in H^+^-ATPase activity on the induction of photosynthetic responses. Thus, the problem of the influence of changes in H^+^-ATPase activity on photosynthesis is the important and requires further investigations. 

Theoretical analysis, using mathematical models of photosynthesis, can be the basis of detailed investigation of the role of different systems of leaf mesophyll, including the conductance of the plasma membrane and chloroplast envelopes, diffusion of CO_2_ in the cytoplasm and apoplast and the activities of systems of ion transport, etc., in photosynthetic regulation. Earlier, we preliminarily used a simple “point model” for analysis of the influence of H^+^-ATPase activity on the ratio of CO_2_ to HCO_3_^−^ in the apoplast, and for estimation of the influence of this activity on photosynthesis [27]; however, using more detailed description of photosynthetic processes and taking into account spatial heterogeneity of a leaf can also be important in theoretical investigation of this problem. 

There are different photosynthetic models (mainly based on the Farquhar—von Caemmerer—Berry model) developed for analysis of mesophyll conductance [3,4,37] and the vertical distribution of CO_2_ in leaf depth [38,39,40]. These models show the role of different leaf structures in mesophyll conductance, and simulate the dependence of CO_2_ assimilation on its distance from the illuminated leaf surface [4,41]. However, analysis of the influence of surface distribution of the photosynthetic processes can also be important for understanding the role of H^+^-ATPase in photosynthetic regulation [42], because lateral propagation of carbon dioxide and its influx into cells can be dependent upon the ratio of CO_2_ to HCO_3_^−^, and on the CO_2_ conductance of the plasma membrane and chloroplast envelopes. Additionally, it is notable that analysis of spatial distribution of the photosynthetic CO_2_ assimilation rate on the leaf surface can show changes in this distribution, which can be markers of electrical signal-induced photosynthetic response [43,44]. Revealing these markers can be important for the development of new methods of analysis of electrical activity in plants, which are actively developing [45,46,47]. 

Earlier, we developed the two-dimensional model of photosynthetic processes in leaves [42], which can be used for investigation of the influence of membrane conductance and H^+^-ATPase activity on the photosynthetic CO_2_ assimilation rate and the spatial heterogeneity of this rate on the leaf surface. The current work has the following specific aims: (i) Analysis of the influence of changes in H^+^-ATPase activity on the photosynthetic CO_2_ assimilation rate. This analysis should show whether H^+^-ATPase activity influences photosynthesis through changes in pH and changes in the ratio of CO_2_ to HCO_3_^−^ (without additional mechanisms). (ii) Analysis of the influence of CO_2_ conductance of the plasma membrane and chloroplast envelopes on the photosynthetic assimilation rate. This problem was important because the influence of electrical signals on the mesophyll CO_2_ conductance was shown earlier [30], and this mechanism should be considered even in the absence of the influence of changes in H^+^-ATPase activity on photosynthesis (at negative response to Question (i)). (iii) Analysis of the influence of both changes in H^+^-ATPase activity and changes in the CO_2_ conductance of the plasma membrane and chloroplast envelopes on the spatial heterogeneity of distribution of the photosynthetic assimilation rate on the leaf surface. Thus, the general objective of the current investigation was further clarification of the mechanisms of influence of electrical signals on photosynthesis in plants, and of the searching markers of this influence.

## 2. Description of the Two-Dimensional Model

**Figure 1:**
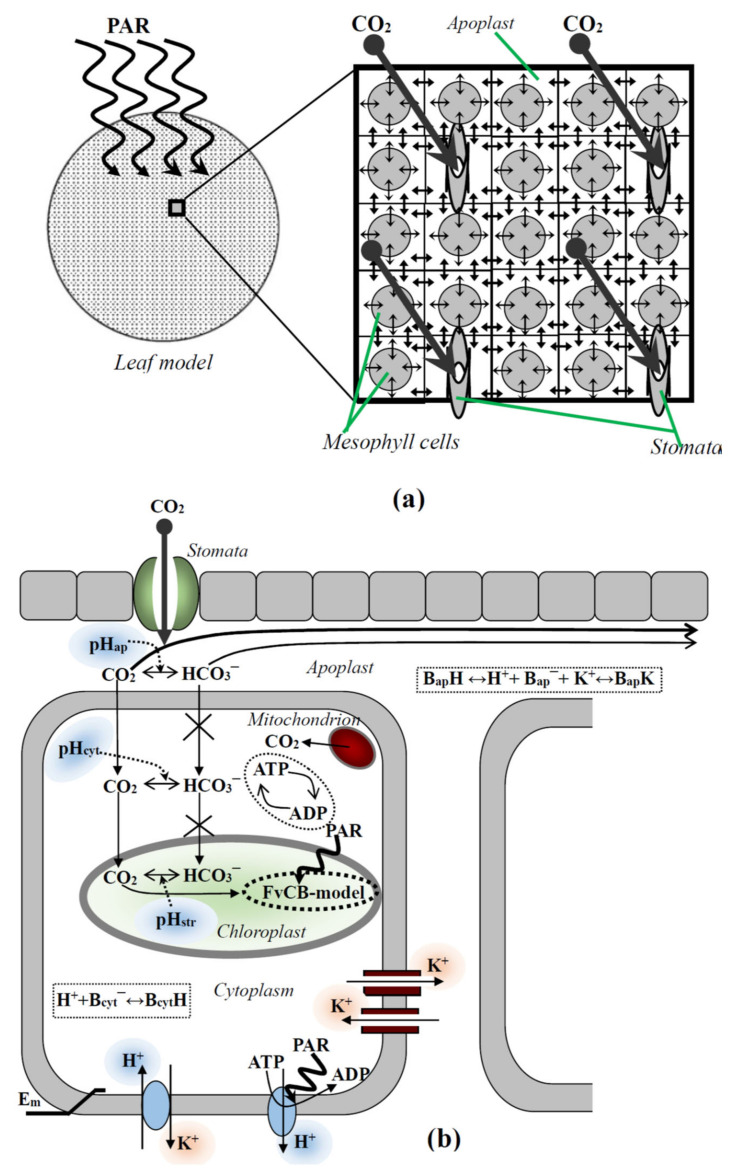
(**a**) A general scheme of the two-dimensional model of photosynthesis in a leaf. The simulated leaf is round and is composed of cells which are connected through the apoplast. Small arrows show transport of carbon dioxide, H^+^, and K^+^ between apoplastic volumes of neighboring cells and across the plasma membrane. PAR is the photosynthetically active radiation. (**b**) A description of ion and CO_2_ fluxes, the activities of ion transporters, buffer capacities, and photosynthetic and respiratory processes simulated by the model. pH_ap_, pH_cyt_, and pH_str_ are the pH in the apoplast, cytoplasm, and stroma of chloroplasts, respectively. B_cyt_^−^ and B_cyt_H are the free and proton-bound cytoplasmic buffers. B_ap_^−^, B_ap_H, and B_ap_K are the free, proton-bound, and potassium-bound apoplastic buffers. E_m_ is the gradient of electrical potential across the plasma membrane. FvCB-model is the Farquhar–von Caemmerer–Berry model. The main systems of ion transport at rest, including H^+^-ATP-ases, H^+^/K^+^-antiporters, inwardly rectifying K^+^ channels, and outwardly rectifying K^+^ channels are described in the two-dimensional photosynthetic model. The schemes from [42], with modifications, are used.

The two-dimensional model of photosynthesis in leaves, which was earlier developed and verified [42], was used in the current investigation. A detailed description of the equations and parameters of this model is shown in File S1 (Supplementary Materials). Briefly, the model included the two-dimensional system of elements which connected with each other through apoplast space (Figure 1a). Each element included the mesophyll cell and section of the apoplast; some elements also included stomata, which are described as the points of CO_2_ entrance.

Each stomata was located in center of a square with an area equal to 3 × 3 elements or 5 × 5 elements. These variants of stomata localization described different stomata densities or partial stomata closure. CO_2_ entered into the leaf through the stomata. After that, carbon dioxide diffused through apoplast along neighboring cells and was transported into the cytoplasm and chloroplast through the plasma membrane and chloroplast envelopes; these fluxes were described on the basis of Fick’s law [3,48] (Figure 1b). There were two forms of the carbon dioxide which were described in the model (CO_2_ and HCO_3_^−^); conversion between CO_2_ and HCO_3_^−^ was regulated by a pH of medium (the apoplast, cytoplasm, or stroma of chloroplast) [26,27]. CO_2_ and HCO_3_^−^ had different constants of diffusion in water and through biological membranes [3]; in the current work, the HCO_3_^−^ transport through the plasma membrane and chloroplast envelopes was assumed to equal zero. We also assumed that only the CO_2_ form was fixed by Rubisco [6,49]. 

The C_3_ photosynthesis and photorespiration were described on the basis of the classical model by Farquhar—von Caemmerer—Berry [6,49]. This model was based on the limitation of stationary photosynthetic CO_2_ assimilation by the rate of photosynthetic electron transport in thylakoid membranes in chloroplasts, or by rate of CO_2_ carboxylation by Rubisco; the minimal rate was assumed to be the photosynthetic rate. The dark respiration rate was assumed to be constant [5,49]. 

**Figure 2:**
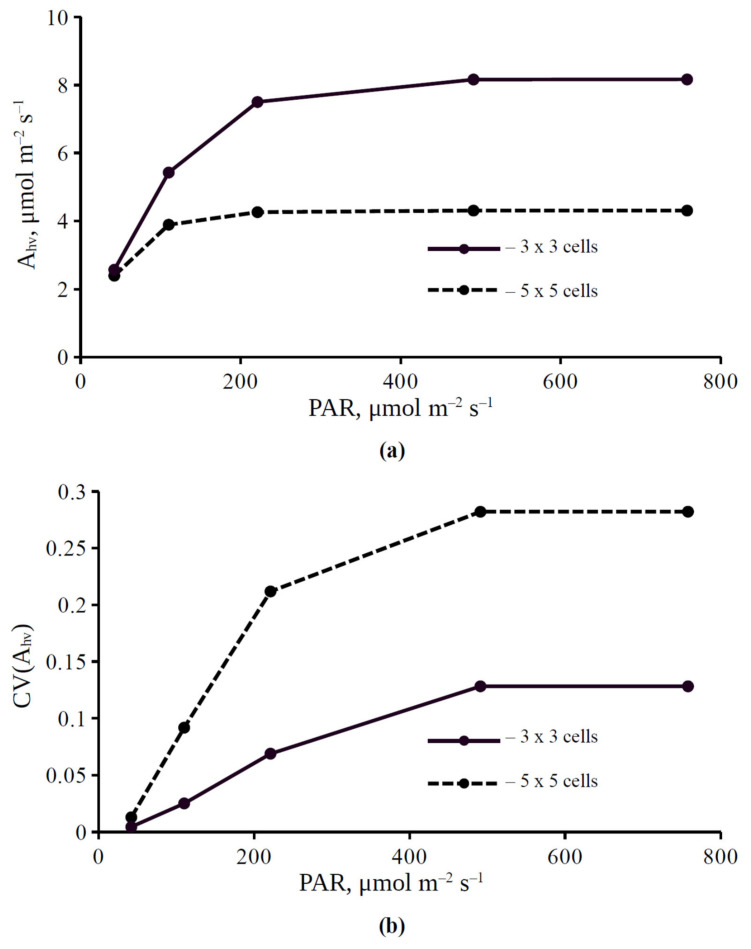
Light dependence of the photosynthetic CO_2_ assimilation rate A_hv_ (**a**) and the coefficient of variation of assimilation CV(A_hv_) (**b**). PAR is photosynthetically active radiation. Each stomata in the simulated leaf was located in center of a 3 × 3 cell square or a 5 × 5 cell square. The basic values of the model parameters were used.

The ion transport (H^+^ and K^+^) and gradient of electrical potential across the plasma membrane were described on the basis of our earlier work [50,51]. In the current work, this description was simplified, and included only H^+^-ATPase, K^+^/H^+^-antiporter and inwardly and outwardly rectifying K^+^-channels. The directions of transport of ions through these transporters are shown in Figure 2b. 

The fluxes through inwardly and outwardly rectifying K^+^-channels were described by the Goldman–Hodgkin–Katz equation [52,53]; the probability of the opening of these channels was described as a function of the electrical potential gradient across the plasma membrane [53,54]. 

The H^+^-ATPase activity was described on the basis of the two-state model of active ion transport, which included the free and proton-bound states of this transporter [53,55,56]. Additionally, regulation of H^+^-ATPase activity by light intensity and ATP concentration was described in the model [57]. The stationary concentration of ATP was described as a function of the rates of photosynthesis, photorespiration and dark respiration.

H^+^ and K^+^ fluxes through the electro-neutral K^+^/H^+^-antiporter were described on the basis of simple equations of chemical kinetics, in accordance with our previous work [54]. 

The diffusion of K^+^ and H^+^ from the apoplast along neighboring cells was described on the basis of Fick’s law [58]. The buffer capacity of H^+^ in the cytoplasm and the capacities of H^+^ and K^+^ in the apoplast were also included into the current model [50,54].

## 3. Results

### 3.1. The Influence of Light Intensity and H^+^-ATPase Activity on Parameters of Photosynthetic Assimilation of CO_2_

In accordance with our previous work [42], influence of light intensity on the photosynthetic CO_2_ assimilation rate and on its spatial heterogeneity on the leaf surface was investigated at the first stage of the current analysis. Light dependence of the CO_2_ assimilation rate and the variation coefficient of this assimilation are shown in Figure 2. It was shown that the CO_2_ assimilation rate and its coefficient of variation (CV(A_hv_)) were increased with increasing photosynthetically active radiation (PAR). The light saturation of these parameters was observed under illumination intensities equal to 500 μmol m^−2^ s^−1^ and more; this value was similar to the light saturation of A_hv_ in pea leaves [42]. It should be noted that the CO_2_ assimilation rate was more in the variant with stomata localized in center of a 3 × 3 cell square than in the variant with stomata localized in center of a 5 × 5 cell square (Figure 2a). In contrast, the coefficient of variation of the CO_2_ assimilation rate was higher in the variant with a 5 × 5 cell square (Figure 2b).

**Figure 3:**
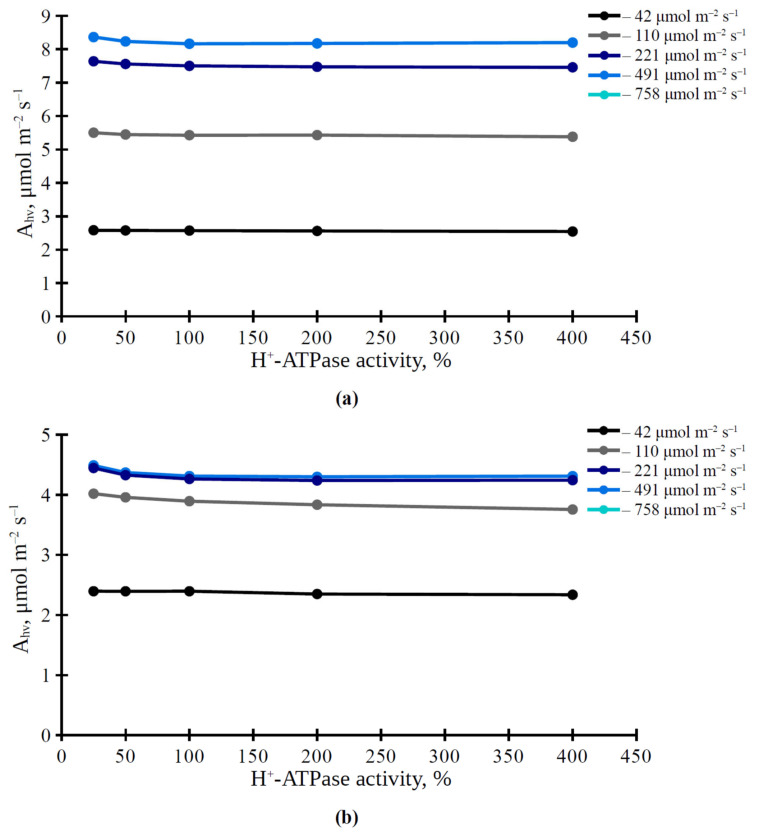
Simulated dependence of the photosynthetic CO_2_ assimilation rate (A_hv_) on the H^+^-ATPase activity in variants with stomata located in center of the 3 × 3 cell square (**a**) or in center of the 5 × 5 cell square (**b**) under various intensities of PAR. The value of the H^+^-ATPase activity from our previous work [42] was used as 100%.

**Figure 4:**
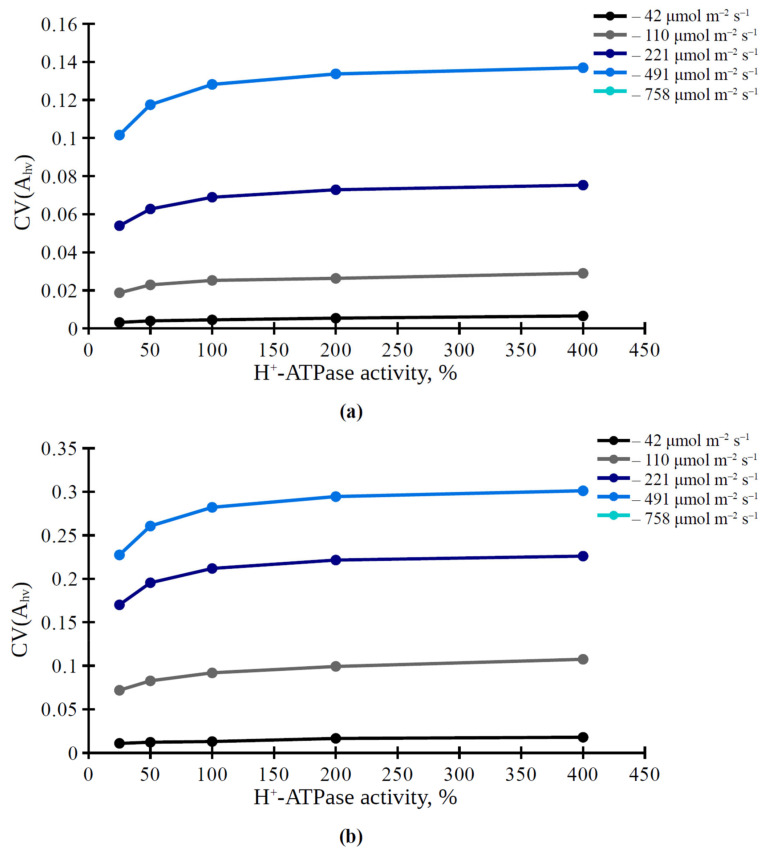
Simulated dependence of variation coefficient of spatial distribution of the photosynthetic CO_2_ assimilation rate (CV(Ahv)) on the H^+^-ATPase activity in variants with stomata located in center of 3 × 3 cells square (**a**) or in center of 5 × 5 cells square (**b**) under various intensities of PAR. Value of the H^+^-ATPase activity from our previous work [42] was used as 100%.

**Figure 5:**
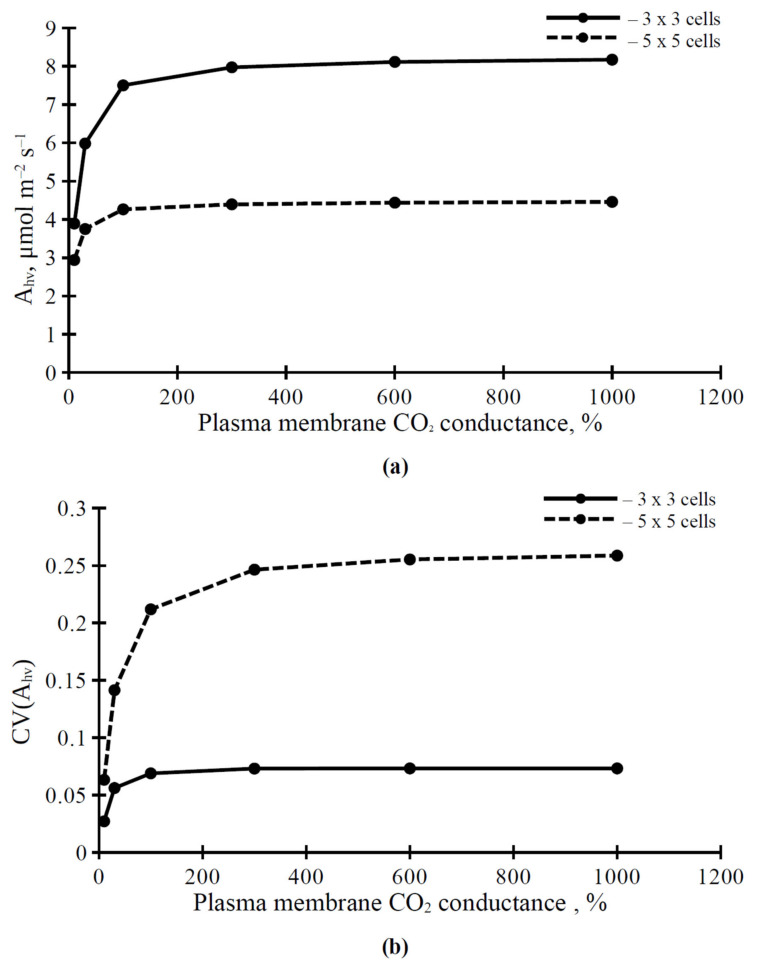
Dependence of the photosynthetic CO_2_ assimilation rate (A_hv_) (**a**) and the coefficient of variation of the spatial distribution of this rate (CV(A_hv_)) (**b**) on the plasma membrane CO_2_ conductance under PAR intensity equaling to 221 μmol m^−2^ s^−1^. Each stomata in the simulated leaf was located in center of a 3 × 3 cell square or in center of a 5 × 5 cell square. The value of the plasma membrane CO_2_ conductance from our previous work [42] was used as 100%.

Furthermore, we analyzed the influence of the H^+^-ATPase activity on the average photosynthetic CO_2_ assimilation rate in the simulated leaf and on the spatial distribution of this rate on the leaf surface. It was shown that A_hv_ and CV(A_hv_) were dependent on light intensity at all levels of activity of H^+^-ATPase in the plasma membrane (Figure 3 and Figure 4). Average A_hv_ in the simulated leaf was weakly dependent on the H^+^-ATPase activity in both investigated variants, 3 × 3 cells (Figure 3a) and 5 × 5 cells (Figure 3b). In contrast, CV(A_hv_) was dependent on H^+^-ATPase activity; increased CV(A_hv_) was observed under the increased H^+^-ATPase activity (Figure 4). This effect was more dramatic under high and medium light intensities. 

### 3.2. The Influence of CO_2_ Conductance of Plasma Membrane and Chloroplast Envelopes on Parameters of Photosynthetic Assimilation of CO_2_

We analyzed the influence of the plasma membrane and chloroplast envelope conductance of CO_2_ on the photosynthetic assimilation rate, and its spatial heterogeneity, in the simulated leaf. It was shown that dependence of A_hv_ (Figure 5a), and CV(A_hv_) (Figure 5b) on the CO_2_ conductance of the plasma membrane was nonlinear. The saturation of this dependence was observed under CO_2_ conductance of the plasma membrane that equaled 300 %, from the initial activity and more (Figure 5). The value of A_hv_ was higher in the 3 × 3 cell variant than in the 5 × 5 cell variant (Figure 5a). In contrast, CV(A_hv_) was higher in the 5 × 5 cell variant than in the 3 × 3 cell variant (Figure 5b).

**Figure 6:**
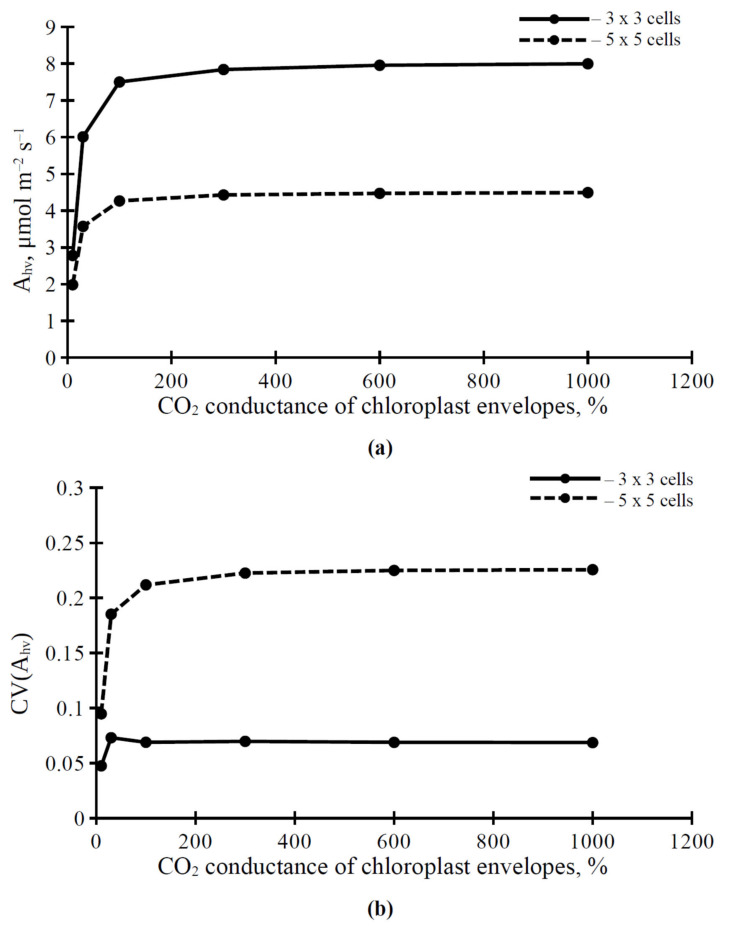
Dependence of the photosynthetic CO_2_ assimilation rate (A_hv_) (**a**) and the coefficient of variation of the spatial distribution of this rate (CV(A_hv_)) (**b**) on the CO_2_ conductance of chloroplast envelopes under PAR intensity equaling to 221 μmol m^−2^ s^−1^. Each stomata in the simulated leaf was located in center of a 3 × 3 cell square or in center of a 5 × 5 cell square. The value of the CO_2_ conductance of chloroplast envelopes from our previous work [42] was used as 100%.

The dependence of A_hv_ and CV(A_hv_) on the CO_2_ conductance of chloroplast envelopes (Figure 6) was similar to the dependence of parameters of the photosynthetic CO_2_ assimilation on the CO_2_ conductance of the plasma membrane (Figure 5). However, there were some differences between these dependences. The values of A_hv_ and CV(A_hv_) under the weak CO_2_ conductance of chloroplast envelopes were lower than the parameters under the similar CO_2_ conductance of the plasma membrane; the increase of A_hv_ and CV(A_hv_) was sharper with an increase in the CO_2_ conductance of chloroplast envelopes (Figure 6). The values of CV(A_hv_) in the 5 × 5 cell variant were lower under the high CO_2_ conductance of chloroplast envelopes (Figure 6b) than the values under the high CO_2_ conductance of the plasma membrane (Figure 5b).

Finally, it was shown that the revealed effects could be also observed in simultaneous changes in the H^+^-ATPase and in the plasma membrane CO_2_ conductance (Table 1 and Table 2). It should be noted that these effects were only checked for the variant with a 3 × 3 cell square (because qualitive differences in the dependence of A_hv_ and CV(A_hv_) in variants with a 3 × 3 cell square and a 5 × 5 cell square were absent in our analysis) and for only the plasma membrane CO_2_ conductance (because the influence of CO_2_ conductance in the plasma membrane and chloroplast envelopes on A_hv_ and CV(A_hv_) was similar).

## 4. Discussion

Photosynthesis is a key process in plants which permits them to assimilate CO_2_ and is the basis of productivity. The photosynthetic assimilation and, therefore, productivity depend on the mesophyll conductance of CO_2_ [20]. The conductance of mesophyll can be modified by environmental factors, physiological processes and the structural parameters of mesophyll cells [21,37,59,60], which can modify the activity of CO_2_ assimilation. The total mesophyll CO_2_ conductance is dependent on the conductance of the cell wall, plasma membrane, cytoplasm, chloroplast envelopes and chloroplast stroma [20,24,61]; it should be noted that the contribution of the plasma membrane and chloroplast envelopes to the total conductance is key.

Photosynthesis is very sensitive to the influence of different environmental factors, including stressors [13]. Long-term changes in photosynthesis can be caused by structural changes in photosynthetic apparatus, synthesis and degradation of photosynthetic pigments [62,63,64] and modification of the mesophyll conductance [13,21,60]. On the other hand, changes in the mesophyll CO_2_ conductance can be also caused by relatively fast processes, including physiological responses induced by systemic action of abiotic environmental factors (e.g., light intensity and temperature [13]), and by local action of stressors causing generation and propagation of specific stress signals (electrical signals) [30]. 

Considering the strong influence of electrical signals on photosynthesis (including suppression of photosynthetic CO_2_ assimilation and linear electron flow, and activation of the cyclic electron flow and non-photochemical quenching of fluorescence of chlorophylls [31,32,33,34]) and the role of these photosynthetic responses in increasing plant tolerance to action of stressors [33,34], analysis of the ways that these signals influence mesophyll CO_2_ conductance is a very important scientific task. This analysis requires understanding of the limiting stages of CO_2_ transport from the intercellular space of the leaf to the chloroplast stroma; these stages are basis of mesophyll CO_2_ conductance and its regulation. Investigation of the limiting stages can be based on investigation of transgenic plants [24], meta-analyses of the literature data showing the mesophyll CO_2_ conductance in different plant species [13,21], and development of mathematical models of CO_2_ transport [3,37]. The latter method seems to be very effective because it can be used for complex investigation of the problem (if necessary, in combination with experimental measurements). 

Earlier, we showed that changes in the H^+^-ATPase activity in the apoplastic and cytoplasmic pH are mechanisms of electrical signals’ influence on photosynthesis [33,34,65]. On the basis of a simulation, it was also shown [27] that the changes in the H^+^-ATPase activity strongly influence the ratio of CO_2_ to HCO_3_^−^, and thereby, should modify transport of CO_2_ into the chloroplast stroma, because charged HCO_3_^−^ is weakly transported through the biological membranes [3,26]. Further analysis of this hypothesis, which was based on the two-dimensional model of photosynthesis in leaves [42], was the first main task of the current work. 

The analysis shows that changes in the H^+^-ATPase activity weakly influence the photosynthetic CO_2_ assimilation rate (Figure 3 and Table 1). It means that our hypothesis [27,33] about the key role of modification of the ratio of CO_2_ to HCO_3_^−^ in the apoplast and cytoplasm—which is caused by changes in the H^+^-ATPase activity and changes in pH in these compartments—in the electrical signals-induced photosynthetic response, seems to be incorrect (i.e., the influence of activity of H^+^-ATPase in the plasma membrane on conversion between CO_2_ and HCO_3_^−^ is not sufficient for regulation of photosynthetic CO_2_ assimilation, at least for the stationary assimilation). In contrast, changes in the CO_2_ conductance of the plasma membrane and chloroplast envelopes, which are the basis of mesophyll CO_2_ conductance, strongly influence A_hv_ (Figure 5a and Figure 6a). This result is in good accordance with an alternative hypothesis about the influence of electrical signals on photosynthesis: ESs-related changes in the H^+^-ATPase activity and changes in the apoplastic and cytoplasmic pH modify the activity of aquaporins [30], which participate in CO_2_ transport through biological membranes in plants and support photosynthetic processes [23,24,25]. It should be noted that we do not directly describe the influence of pH on aquaporin activity to simplify our model; including this dependence in a future model may be useful for revealing more detailed information about changes in the H^+^-ATPase activity and photosynthetic CO_2_ assimilation. 

In our previous work [42], analysis of the model showed that the spatial heterogeneity of the photosynthetic assimilation rate on the leaf surface is strongly dependent on light intensity and stomata density; changes in this heterogeneity (which are estimated on basis of the variation coefficient of spatial distribution of A_hv_ on the leaf surface) can potentially be used for revealing the action of stressors on the plant. It can be expected that changes in the spatial heterogeneity can also be related to the influence of electrical signals on photosynthesis. Thus, analysis of the influence of H^+^-ATPase activity and CO_2_ conductance in the plasma membrane and chloroplast envelopes on the spatial heterogeneity in the CO_2_ assimilation rate on the leaf surface was the second main task of the current work.

We show that CV(A_hv_) is increased with increasing H^+^-ATPase activity (Figure 4) and CO_2_ conductance in the plasma membrane (Figure 5b) and chloroplast envelopes (Figure 6b); this dependence has saturations at high values of the H^+^-ATPase activity and CO_2_ conductance. This effect is also observed in simultaneous changes in H^+^-ATPase activity and CO_2_ conductance (Table 2). Potentially, the CV(A_hv_) increase may be caused (i) by different lateral diffusions of CO_2_ and HCO_3_ (through changes in the ratio between these forms at different levels of H^+^-ATPase activity) and (ii) by the increased carbon dioxide flux into mesophyll cells during the lateral transport of CO_2_ from stomata (through increasing CO_2_ conductance) and, thereby, the increased lateral gradient of CO_2_ concentration in the apoplast. 

The spatial heterogeneity of assimilation (CV(A_hv_)) may be important for monitoring the induction of systemic plant stress responses caused by the propagation of electrical signals [34,66]. It is known that photosynthetic activity is related to optical properties of leaves, including changes in reflectance [67]. The decrease in mesophyll conductance of CO_2_ in particular causes suppression of assimilation [21,37,59,60], which increases ratios of ATP: ADP and NADPH: NADP^+^, stimulates the lumen acidification, and, thereby, suppresses the activity of the electron transport chain of chloroplast [68]. This luminal acidification leads to pigment transformation [69], which changes reflectance in leaves (e.g., by increasing the photochemical reflectance index [70,71]). This means that the development of spatial heterogeneity in photosynthetic activity can lead to the increased spatial heterogeneity in leaf reflectance (e.g., the photochemical reflectance index). Thus, propagation of electrical signals, which are related to changes in the H^+^-ATPase activity (inactivation during the action potential and variation potential [34], activation during the system potential [35]) and influence the mesophyll CO_2_ conductance [30], can potentially be revealed on the basis of spatial heterogeneity in leaf reflectance.

However, the last possibility requires further theoretical and probably experimental analysis, because there are additional factors which can also influence the spatial heterogeneity in the A_hv_. Firstly, our results show that changes in the density of stomata (or changes in the quantity of opened stomata) strongly influence the A_hv_ spatial heterogeneity (Figure 4, Figure 5 and Figure 6). It is known that electrical signals can induce multi-phase responses in stomata (e.g., stomata opening, following their closing) [72,73]. These changes in stomata opening may be an additional factor in CV(A_hv_) changes. Secondly, the parameters of electrical signals can be dependent on their distance from the damaged zone [34], and these parameters are also strongly related to the parameters of photosynthetic response [33]. This means that the propagation of electrical signals can also influence the CV(A_hv_) in leaves; this effect should be more dramatic on large spatial scale. In future, these problems may be solved through the development of a complex model which will include the two-dimensional model of leaf photosynthesis, a model of electrical signal propagation (see, e.g., [53]), and a model of regulation of stomata opening by these signals. 

As a whole, the current investigation shows two main points: (i) The conductance of CO_2_ in the plasma membrane and chloroplast envelopes strongly influences photosynthetic assimilation; in contrast, changes in H^+^-ATPase activity weakly influence this assimilation. (ii) Changes in H^+^-ATPase activity and CO_2_ membrane conductance in the plasma membranes and chloroplast envelopes modify the spatial heterogeneity of the photosynthetic assimilation distribution on the leaf surface. The first result shows that the influence of electrical signals on photosynthesis cannot only be based on changes in the ratio of CO_2_ to HCO_3_^+^, which is dependent on pH and, thereby, the H^+^-ATPase activity. The additional mechanism, of the influence of changes in H^+^-ATPase activity on CO_2_ mesophyll conductance, is necessary. The second result is the basis for the development of new methods of remote sensing of plant systemic responses induced by electrical signals, e.g., methods based on measuring plant reflectance.

## 5. Materials and Methods

We analyzed the two-dimension model of photosynthesis in leaves. The details and equations of the model are described in our previous work [42] and in File S1 (Supplementary Materials). The parameters of the model were estimated on the basis of the literature data (Table S1 in File S1). The verification of the model was performed in our earlier work [42]. The model was numerically analyzed using the forward Euler method in the specialized computer program (Microsoft Visual C++ 2019, Microsoft Corporation, Redmond, WA, USA) developed for solution of this task. 

The analyzed parameters of photosynthesis in leaves were calculated throughout all elements of the simulated leaf. The heterogeneity in the spatial distribution of the photosynthetic assimilation rate was estimated using of the variation coefficient of this rate. 

## 6. Conclusions

The simulated analysis, based on the two-dimensional model of photosynthesis in leaves, showed the following main results: (i) Changes in H^+^-ATPase activity weakly influenced the photosynthetic CO_2_ assimilation rate; i.e., changes in pH and pH-dependent changes in the ratio of CO_2_ to HCO_3_^−^ were not probable to be mechanisms of the influence of electrical signals on photosynthesis. (ii) Decreasing the CO_2_ conductance across the plasma membrane and chloroplast envelopes supressed photosynthetic CO_2_ assimilation; i.e., the decreasing of CO_2_ conductance could be a mechanism of electrical signals’ influence on photosynthesis. (iii) Both changes in the H^+^-ATPase activity and membrane CO_2_ conductance influenced the spatial heterogeneity of the photosynthetic CO_2_ assimilation in the leaf. This result can be used for the development of new methods of estimating the electrical signals in plants, and revealing the physiological responses induced by these signals. 

## Figures and Tables

**Table 1:** Simulated dependence of A_hv_ on the plasma membrane CO_2_ conductance and H^+^-ATPase activity under PAR intensity equal to 221 μmol m^−2^ s^−1^. Each stomata in the simulated leaf was located in center of a 3 × 3 cell square. The values of the H^+^-ATPase activity and plasma membrane CO_2_ conductance from our previous work [42] were used as 100%.

Plasma Membrane Conductance for CO_2_, %	H^+^-ATPase Activity, %				
	25	50	100	200	400
10	3.9173	3.8942	3.8903	3.8810	3.8814
30	6.0721	6.0103	5.9816	5.9794	5.9878
100	7.6391	7.5581	7.5013	7.4731	7.4587
300	8.1319	8.0246	7.9733	7.9631	7.9688
600	8.2778	8.1676	8.1147	8.1039	8.1097
1000	8.3554	8.2256	8.1722	8.1612	8.1669

**Table 2:** Simulated dependence of CV(A_hv_) on simultaneous changes in plasma membrane CO_2_ conductance and H^+^-ATPase activity under PAR intensity equal to 221 μmol m^−2^ s^−1^. Each stomata in the simulated leaf was located in center of a 3 × 3 cell square. The values of the H^+^-ATPase activity and plasma membrane CO_2_ conductance from our previous work [42] were used as 100%.

Plasma Membrane Conductance for CO_2_, %	H^+^-ATPase Activity, %				
	25	50	100	200	400
10	0.021215	0.024805	0.027133	0.028507	0.029249
30	0.044012	0.051382	0.056118	0.058793	0.060186
100	0.053948	0.062738	0.068933	0.072829	0.075273
300	0.05696	0.066834	0.073089	0.076535	0.078442
600	0.057005	0.066942	0.073231	0.076678	0.078577
1000	0.056446	0.066944	0.07324	0.076687	0.078579

## Data Availability

The data presented in this study are available upon request from the corresponding author.

## Supplementary Materials

The following supporting information can be downloaded at: https://www.mdpi.com/article/10.3390/plants11243435/s1, File S1. Description of the two-dimensional photosynthetic model. Table S1. Parameters and initial values of the two-dimensional photosynthetic model. References [74,75,76,77,78] are cited in the Supplementary Materials.
