# Supplementary material for: Simulated Analysis of Influence of Changes in H+-ATPase Activity and Membrane CO2 Conductance on Parameters of Photosynthetic Assimilation in Leaves
Source: Plants (Basel). 2022 Dec 8;11(24):3435. doi: 10.3390/plants11243435 (PMC9783116; doi:10.3390/plants11243435)
Supplement: Supplementary file 1 [file plants-11-03435-s001.zip › plants-2078185-supplementary.pdf]

Supplementary Materials

A mathematical modelling of influence of changes in H^+-ATPase activity and CO_2 conductance in plasma membrane and chloroplast envelopes on photosynthetic assimilation rate and its spatial heterogeneity in leaf

Ekaterina Sukhova [1,*], Daria Ratnitsyna [1] and Vladimir Sukhov [1]

[1] Department of Biophysics, N.I. Lobachevsky State University of Nizhny Novgorod, 603950 Nizhny Novgorod, Russia

* Correspondence: n.catherine@inbox.ru; Tel.: +7-929-040-2938

Description of the two-dimensional photosynthetic model

1. Description of photosynthetic assimilation of CO_2 and photorespiration

The photosynthetic assimilation of CO_2 was described by classical model of Farquhar, von Caemmerer and Berry (FvCB-model) [1-2] with simplifications. The assimilation rate was calculated as minimum between the assimilations rate at the Rubisco carboxylation-limited conditions (W_c) and the assimilations rate at the electron transport rate-limited conditions (W_j):

$$A_{hv} = \min(W_c, W_j)\frac{[CO_2]_{str} - \Gamma^*}{[CO_2]_{str}} \quad \text{(S1)},$$

where $[CO_2]_{str}$ was concentration of CO_2 in the stroma of chloroplasts, Γ^* was the photosynthetic CO_2 compensation point at the absence of mitochondrial respiration. W_c and W_j were described by Equations (S1) and (S2), respectively:

$$W_c = \frac{V_{max}[CO_2]_{str}}{[CO_2]_{str} + K_C\left(1 + \frac{[O_2]}{K_O}\right)} \quad \text{(S2)},$$

$$W_j = \frac{J}{4 + 8\frac{\Gamma^*}{[CO_2]_{str}}} \quad \text{(S3)},$$

where V_{max} was the maximum rate of carboxylation of Rubisco, K_C and K_O were the Michaelis constants for carboxylation and oxygenation, respectively, $[O_2]$ was concentration of O_2 in the stroma of chloroplasts, J was the potential flux of electron through electron transport chain in chloroplast (Equation (S4)):

$$J = \frac{I + J_{max}}{2\theta} - \frac{\sqrt{(I + J_{max})^2 - 4\theta I J_{max}}}{2\theta} \quad \text{(S4)},$$

where I (Equation (S5)) was the useful light absorbed by the photosystem II, J_{max} was the maximum electron transport rate, and θ was an empirical curvature factor [1].

$$I = \frac{abs(1 - f)}{2} PAR \quad \text{(S5)},$$

where abs was the light absorptance by chlorophyll, and f was the factor of correction for spectral quality of the light for absorbance by chlorophyll, PAR was the photosynthetically active radiation.

The photorespiration rate (V_{phr}) was calculated by Equation (S6):

$$V_{phr} = \frac{(\min(W_c, W_j) + R_d)\Gamma^*}{[CO_2]_{str}} \quad (S6),$$

The rate of the dark respiration (R_d) was assumed constant in accordance with von Caemmerer et al. [1].

2. Description of stomata and transport of CO_2 through mesophyll

The transport of CO_2 through the stomata (j_S), through the plasma membrane (j_{PM}), and through the chloroplast envelopes (j_{Chl}) were described by using of the Fick's law [3]:

$$j_S = g_S{}^0([CO_2]_{out} - [CO_2]_{ap}) \quad (S7),$$

$$j_{PM} = g_{PM}([CO_2]_{ap} - [CO_2]_{cyt}) \quad (S8),$$

$$j_{Chl} = g_{Chl}([CO_2]_{cyt} - [CO_2]_{str}) \quad (S9),$$

where $[CO_2]_{out}$, $[CO_2]_{ap}$, and $[CO_2]_{cyt}$ were concentrations of CO_2 in the air, apoplast and cytoplasm, respectively, $g_S{}^0$, g_{PM}, and g_{Chl} were CO_2 conductance for the stomata, plasma membrane, and chloroplast envelopes, respectively.

The conductance of stomata (g_S) was assumed constant. It was also assumed that the CO_2 conductance per area unit was equal for the plasma membrane and chloroplast envelopes [4-5]. Thus, the CO_2 conductance of mesophyll (g_m) was related with g_{PM} and g_{Chl} as:

$$g_{Chl} = \frac{g_m\left(1 + \frac{S_{PM}}{S_{Chl}}\right)}{\frac{S_{PM}}{S_{Chl}}} \quad (S10),$$

$$g_{pm} = g_m\left(1 + \frac{S_{PM}}{S_{Chl}}\right) \quad (S11),$$

where $\frac{S_{PM}}{S_{Chl}}$ was ratio of the total area of the plasma membranes to the total area of the envelopes of chloroplasts.

3. Description of diffusion of CO_2 and HCO_3^- through apoplast

The volume fluxes of CO_2 ($j_{CO2}{}^{n,k/l,m}$) and HCO_3^- ($j_{HCO3}{}^{n,k/l,m}$) between apoplasts of neighboring cells (coordinates of neighboring cells were marked by indices n, k and l, m) were described by Equations (S12) and (S13) in accordance with our previous work [6]:

$$j_{CO2}{}^{n,k/l,m} = \frac{D_{CO2}}{a^2(1 + \frac{V_{ap}}{V_{cell}})^{1/3}}([CO_2]_{ap}{}^{l,m} - [CO_2]_{ap}{}^{n,k}) \quad (S12),$$

$$j_{HCO3}{}^{n,k/l,m} = \frac{D_{HCO3}}{a^2(1 + \frac{V_{ap}}{V_{cell}})^{1/3}}([HCO_3{}^-]_{ap}{}^{l,m} - [[HCO_3{}^-]]_{ap}{}^{n,k}) \quad (S13),$$

where a was linear size of cube cell, D_{CO2} and D_{HCO3} were diffusion coefficients of CO_2 and HCO_3^- in water, $[HCO_3^-]_{ap}$ was concentration of HCO_3^- in the apoplast, and $\frac{V_{ap}}{V_{cell}}$ was ratio of the apoplastic volume to the total cell volume. Fluxes of CO_2 and HCO_3^- in border elements which were directed outside of the simulated leaf were assumed equaling to zero.

4. Description of changes in concentrations of CO_2 and HCO_3^-

Changes in CO_2 and HCO_3^- concentrations in the apoplast (C_{ap}), cytoplasm (C_{cyt}), and chloroplast stroma (C_{str}) were related to transport of CO_2 and HCO_3^- through apolasts, transport of CO_2 through membranes, and rates of the photosynthetic CO_2 assimilation, photorespiration, and respiration (Equations (S14)-(S16)):

$$\frac{dC_{ap}{}^{n,k}}{dt} = 18 \cdot 10^{-5} \frac{S_{leaf}}{V_{ap}} (j_S{}^{n,k} - j_{PM}{}^{n,k}) + j_{CO2}{}^{n,k/n-1,k} + j_{CO2}{}^{n,k/n+1,k} + j_{CO2}{}^{n,k/n,k-1} + + j_{CO2}{}^{n,k/n,k+1} + j_{HCO3}{}^{n,k/n-1,k} + j_{HCO3}{}^{n,k/n+1,k} + j_{HCO3}{}^{n,k/n,k-1} + j_{HCO3}{}^{n,k/n,k+1} \quad (S14),$$

$$\frac{dC_{cyt}{}^{n,k}}{dt} = 18 \cdot 10^{-5} \frac{S_{leaf}}{V_{cyt}} (j_{PM}{}^{n,k} - j_{Chl}{}^{n,k} + R_d + V_{phr}) \quad (S15),$$

$$\frac{dC_{str}{}^{n,k}}{dt} = 18 \cdot 10^{-5} \frac{S_{leaf}}{V_{str}} (j_{Chl}{}^{n,k} - \min(W_c, W_j)) \quad (S16),$$

where $\frac{S_{leaf}}{V_{ap}}$, $\frac{S_{leaf}}{V_{cyt}}$, and $\frac{S_{leaf}}{V_{str}}$ were ratios of leaf areas to volumes of the apoplast, cytoplasm, and stroma, respectively, $18 \cdot 10^{-5}$ dm^3 mol^{-1} was volume of 1 mol of H_2O.

We calculated portion of CO_2 in the summary concentration of CO_2 and HCO_3^- (P_{CO2}) in accordance with our previous work [7]:

$$P_{CO_2} = \frac{1}{1 + 10^{pH-pK}} \quad (S17),$$

where pK was the negative logarithm of the equilibrium constant in the reaction of transition between CO_2 and HCO_3^-. Thus, the final CO_2 concentrations were calculated as multiplication of P_{CO2} and the summary concentration of CO_2 and HCO_3^-; the final HCO_3^- concentrations were calculated as multiplication of (1-P_{CO2}) and the summary concentration of CO_2 and HCO_3^-.

5. Description of ion transport though plasma membrane and membrane potential

H^+ and K^+ fluxes through the plasma membrane was described in accordance with our previous model [8-9]; however, this model was simplified and included only H^+-ATPase, inwardly and outwardly rectifying K^+ channels, and K^+/H^+-antiporter.

A proton flux through H^+-ATPase (j_P) was described by the "two-state model" [8, 10]:

$$j_P = A_{ATP} A_{BL} \frac{k_{+1}k_{+2} - k_{-1}k_{-2}}{k_{+1} + k_{+2} + k_{-1} + k_{-2}} \quad (S18),$$

where $[H^+]_{ap}$ and $[H^+]_{cyt}$ were proton concentrations in the apoplast and cytoplasm, respectively, $k_{+1} = k_1[H^+]_{ap}$, $k_{-1} = k_1 \exp\left(\frac{G_{ATP}}{RT}\right)$, $k_{+2} = \frac{k_2 u}{1-\exp(-u)}$, and $k_{-2} = \frac{k_2 u [H^+]_{cyt} \exp(-u)}{1-\exp(-u)}$ were velocity constants for transitions between states of the H^+-ATPase, k_1 and k_2 were are velocity constants of transitions between states of the H^+-ATPase at u=0 and $[H^+]_{ap} = [H^+]_{out} = 1$ M, $u = \frac{E_m F}{RT}$ was the normalized membrane potential, G_{ATP} was the energy of ATP hydrolysis, E_m was membrane potential across the plasma membrane, F, R, and T were standard thermodynamic values, A_{ATP} (Equation (S19)) and A_{BL} (Equation (S20)) were coefficients describing activation of H^+-ATPase by the cytoplasmic ATP concentration ([ATP]) and intensity of blue light (BL), respectively.

$$A_{ATP} = \frac{K_{cyt}[ATP]}{K_{ATP} + K_{cyt}[ATP]} \quad (S19),$$

$$A_{BL} = \frac{BL}{BL + K_{BL}} + A_{BL0} \frac{K_{BL}}{K_{BL} + BL} \quad (S20),$$

where K_{ATP} was the constant of the 50% activation of H^+-ATPase by [ATP], K_{cyt} was the proportional coefficient between concentrations of ATP in the leaf and cytoplasm, A_{BL0} was activity of H^+-ATPase without the blue light, K_{BL} was the constant of the 50% activation of H^+-ATPase by BL.

The Goldman–Hodgkin–Katz equation was used for description of K^+ fluxes through inwardly (J_{IRKC}) and outwardly (J_{ORKC}) rectifying K^+ channels [8-9, 11]:

$$J_{IRKC} = \frac{P_{IRKC} P_{max}{}^{IRKC} u \left([K^+]_{cyt} - [K^+]_{ap} \exp(-u)\right)}{1 - \exp(-u)} \quad \text{(S21)},$$

$$J_{ORKC} = \frac{P_{ORKC} P_{max}{}^{ORKC} u \left([K^+]_{cyt} - [K^+]_{ap} \exp(-u)\right)}{1 - \exp(-u)} \quad \text{(S22)},$$

where P_{IRKC} (Equation (S23)) and P_{ORKC} (Equation (S24)) were probabilities of open states of inwardly and outwardly rectifying K^+ channels, respectively, where $P_{max}{}^{IRKC}$ and $P_{max}{}^{ORKC}$ were maximum permeabilities of inwardly and outwardly rectifying K^+ channels, respectively, $[K^+]_{cyt}$ and $[K^+]_{ap}$ were concentrations of K^+ in the cytoplasm and apoplast.

$$P_{IRKC} = \frac{1}{1 + \exp\left(c_{IRKC}(u - u_{IRKC})\right)} \quad \text{(S23)},$$

$$P_{IRKC} = \frac{1}{1 + \exp\left(c_{IRKC}(u_{IRKC} - u)\right)} \quad \text{(S24)},$$

where c_{IRKC} and c_{ORKC} were constants which represented a portion of the membrane potential acting on the gating mechanisms and their charge in inwardly and outwardly rectifying K^+ channels, respectively, u_{IRKC} and u_{ORKC} were the normalized potential barriers for the transition of the channel from the closed state to the open one in inwardly and outwardly rectifying K^+ channels, respectively.

The transport of H^+ and K^+ through K^+/H^+-antiporter (J_{Ant}) were calculated in accordance with our previous works [8-9]:

$$J_{Ant} = k_{Ant}\left([K^+]_{cyt}[H^+]_{ap} - [K^+]_{ap}[H^+]_{cyt}\right) \quad \text{(S25)},$$

where k_{Ant} was parameter which was proportional to rate of transports of ions through the antiporter.

We used stationary value of membrane potential E_m which was calculated in accordance with our previous works [6, 9]:

$$E_m = \frac{g_K E_K + g_P E_P}{g_K + g_P} \quad \text{(S26)},$$

where E_K and E_P were reverse potentials for K^+ channels (Equation (S27)) and H^+-ATPase (Equation (S28)), respectively, g_K and g_P were electrical conductance for K^+ channels (Equation (S29)) and H^+-ATPase (Equation (S30)), respectively:

$$E_K = \frac{RT}{F} \ln\left(\frac{[K^+]_{ap}}{[K^+]_{cyt}}\right) \quad \text{(S27)},$$

$$E_P = \frac{RT}{F} \ln\left(\frac{[H^+]_{ap}}{[H^+]_{cyt}}\right) + \frac{G_{ATP}}{F} \quad \text{(S28)},$$

$$g_K = \frac{F\left(J_{kg} + J_{kd}\right)}{E_m - E_K} \quad \text{(S29)},$$

$$g_P = \frac{FJ_P}{E_m - E_P} \quad \text{(S30)},$$

6. Description of diffusion of H+ and K+ through apoplast

The diffusion K^+ ($j_K^{n,k/l,m}$) and H^+ ($j_H^{n,k/l,m}$) between apoplasts of two neighboring cells (their coordinates were marked by indices n, k and l, m) were described in accordance with our previous work [6]:

$$j_K^{n,k/l,m} = \frac{D_K}{a^2(1+\frac{V_{ap}}{V_{cell}})^{1/3}}([K^+]_{ap}{}^{l,m} - [K^+]_{ap}{}^{n,k}) \quad \text{(S31)},$$

$$j_H^{n,k/l,m} = \frac{D_H}{a^2(1+\frac{V_{ap}}{V_{cell}})^{1/3}}([H^+]_{ap}{}^{l,m} - [[H^+]]_{ap}{}^{n,k}) \quad \text{(S32)},$$

where D_K and D_H were coefficients of diffusion of K^+ and H^+ in water. Ion fluxes directed outside in border cells of the simulated leaf were assumed equaling to zero.

7. Description of changes in K+ and H+ concentrations

Changes in total K^+ concentrations in the apoplast ($[K]_{ap}$) and cytoplasm ($[K^+]_{cyt}$) and changes in total H^+ concentrations in the apoplast ($[H]_{ap}$) and cytoplasm ($[H]_{cyt}$), respectively, which were related with membrane transporters, were described by Equations (S33-S36):

$$\frac{d[K]_{ap}{}^{n,k}}{dt} = \frac{1}{a}\left(\frac{V_{ap}}{V_{cell}}\right)^{-1}(j_{IRKC}{}^{n,k} + j_{ORKC}{}^{n,k} + j_{Ant}{}^{n,k}) + j_K{}^{n,k/n-1,k} + j_K{}^{n,k/n+1,k} + j_K{}^{n,k/n,k-1} + j_K{}^{n,k/n,k+1} \quad \text{(S33)},$$

$$\frac{d[K^+]_{cyt}{}^{n,k}}{dt} = -\frac{1}{a}\left(\frac{V_{cyt}}{V_{cell}}\right)^{-1}(j_{IRKC}{}^{n,k} + j_{ORKC}{}^{n,k} + j_{Ant}{}^{n,k}) \quad \text{(S34)},$$

$$\frac{d[H]_{ap}{}^{n,k}}{dt} = \frac{1}{a}\left(\frac{V_{ap}}{V_{cell}}\right)^{-1}(j_P{}^{n,k} - j_{Ant}{}^{n,k}) + j_H{}^{n,k/n-1,k} + j_H{}^{n,k/n+1,k} + j_H{}^{n,k/n,k-1} + j_H{}^{n,k/n,k+1} \quad \text{(S35)},$$

$$\frac{d[H]_{cyt}{}^{n,k}}{dt} = -\frac{1}{a}\left(\frac{V_{cyt}}{V_{cell}}\right)^{-1}(j_P{}^{n,k} - j_{Ant}{}^{n,k}) \quad \text{(S36)},$$

where $\frac{V_{cyt}}{V_{cell}}$ was ratio of the cytoplasmic volume to the total cell volume.

The influence of buffer capacity of the cytoplasm for H^+ and buffer capacity of the apoplast for K^+ and H^+ [8-9] were described by Equation (S37) and (S38), respectively. Concentration of H^+ in stroma ($[H^+]_{str}$) was assumed as constant.

$$[H^+]_{cyt} = \frac{([H]_{cyt} - B_{cyt} - K_H{}^{cyt})}{2} + \frac{\sqrt{([H]_{cyt} - B_{cyt} - K_H{}^{cyt})^2 + 4K_H{}^{cyt}[H]_{cyt}}}{2} \quad \text{(S37)},$$

$$[H^+]_{ap} = \frac{-K_H{}^{ap}[H]_{ap}(B_{ap}-[H]_{ap}-[K]_{ap}-K_K{}^{ap})}{2(K_K{}^{ap})(B_{ap}-[H]_{ap})} + \frac{\sqrt{(K_H{}^{ap}[H]_{ap}(B_{ap}-[H]_{ap}-[K]_{ap}-K_K{}^{ap}))^2 + 4K_K{}^{ap}(K_H{}^{ap})^2([H]_{ap})^2(B_{ap}-[H]_{ap})}}{2K_K{}^{ap}(B_{ap}-[H]_{ap})} \quad \text{(S38)},$$

$$[K^+]_{ap} = \frac{K_K{}^{ap}[H^+]_{ap}[K]_{ap}}{K_H{}^{ap}[H]_{ap} + K_K{}^{ap}[H^+]_{ap}} \quad \text{(S39)},$$

where B_{cyt} and B_{apt} were total concentration of proton buffer (free and bonded) in the cytoplasm and apoplast, respectively, K_H^{cyt} was the dissociation constant between the cytoplasmic buffer and H^+, K_H^{ap} and K_K^{ap} were the dissociation constants between the apoplastic buffer and H^+ and between the apoplastic buffer and K^+.

8. Description of changes in ATP concentration

Stationary concentration of ATP ([ATP]) per unit of the leaf volume was described by Equation (S40):

$$[ATP] = \frac{\beta(R_d + \alpha \cdot \min(W_c, W_j))}{\beta\left(R_d + \alpha \cdot \min(W_c, W_j)\right) + k_{cons}} ATP_\Sigma \qquad (S40),$$

where $\beta = \frac{S_{leaf}}{V_{leaf}} \frac{5}{ATP_\Sigma - [ATP]_{dark}}$ was constant, $\frac{S_{leaf}}{V_{leaf}}$ was ratio of the leaf area to the leaf volume, ATP_Σ and $[ATP]_{dark}$ were total concentration of ATP and ADP and concentration of ATP under dark conditions (without photosynthetic processes), α was portion of the CO_2 assimilation rate which was used for the ATP synthesis, and k_{cons} was effective velocity constant of all processes of the ATP consumption (this parameter was calculated on basis of only Rd under dark conditions).

9. Model parameterization

Table S1 shows main parameters and initial values of ion concentration which were used in the work. It should be noted that initial concentrations of HCO_3^- and initial summary concentrations of CO_2 and HCO_3^- were calculated on basis of initial concentrations of CO_2 and equation (S17).

Table S1. Parameters and initial values of the **two**-dimensional photosynthetic model

Parameters	*Values*	*Units*	*Sources for calculation of values*
Photosynthetic CO_2 assimilation and photorespiration			
**K_C**	260	ppm	[1]
**K_O**	179000	ppm	[1]
**V_{max}**	80	μmol m^{-2} s^{-1}	[1]
**Γ^***	38.6	ppm	[1]
**$[O_2]$**	200000	ppm	[1]
**J_{max}**	160	μmol m^{-2} s^{-1}	[1]
**abs**	0.85		[1]
**f**	0.15		[1]
**θ**	0.7		[1]
**R_d**	1	μmol m^{-2} s^{-1}	[1]
Stomata and transmembrane CO_2 fluxes			
**$[CO_2]_{out}$**	360	ppm	Assumed
**g_S^0 (model elements with both mesophyll cell and stomata)**	0.576 (basic) or 0.207 (decreased)	mol m^{-2} s^{-1}	The basic g_S^0 was calculated as $g_S \cdot 9$, where $g_S = 0.064$ mol m^{-2} s^{-1} (the current experiment). The decreased g_S^0 was calculated as 0.576·9/25
**g_S^0** (**model elements without stomata**)	0	mol m^{-2} s^{-1}	Assumed

g_m	0.1	mol m^{-2} s^{-1}	[12]
$\frac{S_{PM}}{S_{Chl}}$	0.495		[4-5]

Lateral CO_2 and HCO_3^- fluxes

**a**	10^{-3}	dm	[6]
D_{CO2}	$1.83 \cdot 10^{-7}$	dm^2 s^{-1}	[5]
D_{HCO3}	$0.95 \cdot 10^{-7}$	dm^2 s^{-1}	[5]
$\frac{V_{ap}}{V_{cell}}$	0.1		[11]

Changes in CO_2 and HCO_3^- concentrations

$\frac{S_{leaf}}{V_{ap}}$	5500	dm^{-1}	[11, 13]
$\frac{S_{leaf}}{V_{cyt}}$	13750	dm^{-1}	[3, 11, 13]
$\frac{S_{leaf}}{V_{str}}$	6790	dm^{-1}	[3, 11, 13]
**pK**	6.35		[7]

Description of transmembrane H^+ and K^+ fluxes and membrane potential

	H^+-ATPase		
k_1	0.045	s^{-1}	[9]
k_2	$2.58 \cdot 10^{-5}$	s^{-1}	[9]
**T**	296	K	[8]
**F**	96500	C mol^{-1}	[8]
**R**	8.31	J mol^{-1} K^{-1}	[8]
G_{ATP}	-50000	J	[8]
K_{ATP}	$186 \cdot 10^{-6}$	M	[14]
K_{cyt}	5.676		[3]
A_{BL0}	0.566		[14]
K_{BL}	6.55	μmol $m^{-2}s^{-1}$	[14]
	Inwardly and outwardly rectifying K^+ channels		
P_{max}^{IRKC}	$2.9 \cdot 10^{-7}$	dm s^{-1}	[9]
P_{max}^{ORKC}	$2.9 \cdot 10^{-7}$	dm s^{-1}	[9]
u_{IRKC}	-7.4		[8]
u_{ORKC}	-2.53		[8]
c_{IRKC}	1.1		[8]
c_{ORKC}	1.13		[8]
	K^+/H^+-antiporter		
k_{ant}	0.015	M^{-1} s^{-1}	[8]

Description of lateral H^+ and K^+ fluxes

D_H	$7.8 \cdot 10^{-7}$	dm^2 s^{-1}	[6]
D_K	$1.96 \cdot 10^{-7}$	dm^2 s^{-1}	[6]

Description of changes in K^+ and H^+ concentrations

$\frac{V_{cyt}}{V_{cell}}$	0.04		[3]
B_{cyt}	0.2	M	[8]
K_H^{cyt}	10^{-6}	M	[8]
B_{ap}	0.083	M	[8]
K_H^{ap}	10^{-6}	M	[8]
K_K^{ap}	10^{-4}	M	[8]
$[H^+]_{str}$	$3.16 \cdot 10^{-8}$	M	[15]

Description of changes in ATP concentration

α	0.2		Assumed
ATP_Σ	$0.132 \cdot 10^{-3}$	M	[16]
$[ATP]_{dark}$	$0.065 \cdot 10^{-3}$	M	[16]
k_{cons}	0.3846	s^{-1}	Assumed
$\frac{S_{leaf}}{V_{leaf}}$	5000	$m^2\ m^{-3}$	[13]

Initial values of main variables

$[CO_2]_{ap}$	360	ppm	Assumed
$[CO_2]_{cyt}$	360	ppm	Assumed
$[CO_2]_{str}$	360	ppm	Assumed
$[K]_{ap}$ / $[K^+]_{ap}$	$8.2 \cdot 10^{-2}$ / $3.5 \cdot 10^{-3}$	M	[8]
$[K^+]_{cyt}$	$1.4 \cdot 10^{-1}$	M	[17]
$[H]_{ap}$ / $[H^+]_{ap}$	$2 \cdot 10^{-3}$ / 10^{-6}	M	[8]
$[H]_{cyt}$ / $[H^+]_{cyt}$	$1.5 \cdot 10^{-2}$ / $7 \cdot 10^{-8}$	M	[8]

References for Supplementary Materials

1. von Caemmerer, S.; Farquhar, G.; Berry, J. Biochemical model of C_3 photosynthesis, In *Photosynthesis in silico. Advances in Photosynthesis and Respiration.* Laisk, A., Nedbal, L., Govindjee, Eds. Springer, Dordrecht, Germany, 2009; Volume 29, pp. 209-230.
2. Bernacchi, C.J.; Rosenthal, D.M.; Pimentel, C.; Long, S.P.; Farquhar, G.D. Modeling the temperature dependence of C_3. In *Photosynthesis in silico. Advances in Photosynthesis and Respiration*. Laisk, A., Nedbal, L., Govindjee Eds. Springer, Dordrecht, Germany, 2009; Volume 29, pp. 231-246.
3. Winter, H.; Robinson, D.G.; Heldt, H.W. Subcellular volumes and metabolite concentrations in spinach leaves. *Planta*. **1994**, *193*, 530-535.
4. Evans, J.R.; Kaldenhoff, R.; Genty, B.; Terashima, I. Resistances along the CO_2 diffusion pathway inside leaves. *J. Exp. Bot.* **2009**, *60*, 2235–2248.
5. Tholen, D.; Zhu, X.-G. The mechanistic basis of internal conductance: a theoretical analysis of mesophyll cell photosynthesis and CO_2 diffusion. *Plant Physiol.* **2011**, *156*, 90-105.
6. Sukhov, V.; Nerush, V.; Orlova, L.; Vodeneev, V. Simulation of action potential propagation in plants. *J. Theor. Biol.* **2011**, *291*, 47-55.
7. Sukhova, E.M.; Sukhov, V.S. Dependence of the CO_2 uptake in a plant cell on the plasma membrane H^+-ATPase activity: theoretical analysis. *Biochem. Moscow Suppl. Ser. A.* **2018**, *12*, 146-159.
8. Sukhov, V.; Vodeneev, V. A mathematical model of action potential in cells of vascular plants. *J. Membr. Biol.* **2009**, *232*, 59–67.
9. Sukhova, E.; Ratnitsyna, D.; Sukhov, V. Stochastic spatial heterogeneity in activities of H^+-ATP-ases in electrically connected plant cells decreases threshold for cooling-induced electrical responses. *Int. J. Mol. Sci.* **2021**, *22*, 8254.
10. Sukhova, E.; Akinchits, E.; Sukhov, V. Mathematical models of electrical activity in plants. *J. Membr. Biol.* **2017**, *250*, 407-423.
11. Gradmann, D. Impact of apoplast volume on ionic relations in plant cells. *J. Membr. Biol.* **2001**, *184,* 61–69.
12. Flexas, J.; Barbour, M.M.; Brendel, O.; Cabrera, H.M.; Carriquí, M.; Díaz-Espejo, A.; Douthe, C.; Dreyer, E.; Ferrio, J.P.; Gago, J.; Gallé, A.; Galmés, J.; Kodama, N.; Medrano, H.; Niinemets, Ü.; Peguero-Pina, J.J.; Pou, A.; Ribas-

Carbó, M.; Tomás, M.; Tosens, T.; Warren, C.R. Mesophyll diffusion conductance to CO_2: an unappreciated central player in photosynthesis. *Plant Sci.* **2012**, *193-194*, 70-84.
13. Day, T.A.; Vogelmann, T.C. Alterations in photosynthesis and pigment distributions in pea leaves following UV-B exposure. *Physiol. Plant*. **1995**, *94*, 433-440.
14. Kinoshita, T.; Shimazaki, K. Blue light activates the plasma membrane H^+-ATPase by phosphorylation of the C-terminus in stomatal guard cells. *EMBO J.* **1999**, *18*, 5548–5558.
15. Antal, T.K.; Kovalenko, I.B.; Rubin, A.B.; Tyystjärvi, E. Photosynthesis-related quantities for education and modeling. *Photosynth Res*. **2013**, *117*, 1-30.
16. Roeske C.A.; Chollet R. Role of metabolites in the reversible light activation of pyruvate, orthophosphate dikinase in *Zea mays* mesophyll cells *in Vivo*. *Plant Physiol*. **1989**, *90*, 330-337.
17. Wang, Y.; Wu, W.H. Plant sensing and signaling in response to K^+-deficiency. *Mol. Plant.* **2010**, *3*, 280-287.
